# DNA Methylation of Genes Participating in Hepatic Metabolisms and Function in Fetal Calf Liver Is Altered by Maternal Undernutrition during Gestation

**DOI:** 10.3390/ijms241310682

**Published:** 2023-06-26

**Authors:** Susumu Muroya, Konosuke Otomaru, Kazunaga Oshima, Ichiro Oshima, Koichi Ojima, Takafumi Gotoh

**Affiliations:** 1 Division of Animal Products Research, NARO Institute of Livestock and Grassland Science (NILGS), Tsukuba 305-0901, Ibaraki, Japan; koojima@affrc.go.jp; 2 Joint Faculty of Veterinary Medicine, Kagoshima University, Korimoto 1-21-24, Kagoshima 890-8580, Kagoshima, Japan; otomaru@vet.kagoshima-u.ac.jp; 3 Division of Year-Round Grazing Research, NARO Western Region Agricultural Research Center, 60 Yoshinaga, Ohda 694-0013, Shimane, Japan; tenpoint@affrc.go.jp; 4 Department of Agricultural Sciences and Natural Resources, Kagoshima University, Korimoto 1-21-24, Kagoshima 890-8580, Kagoshima, Japan; oshima@agri.kagoshima-u.ac.jp; 5 Field Science Center for Northern Biosphere, Hokkaido University, N11W10, Kita, Sapporo 060-0811, Hokkaido, Japan; gotoh@fsc.hokudai.ac.jp

**Keywords:** DNA methylation, epigenetics, fetal growth restriction, liver, maternal undernutrition, mitophagy, RRBS

## Abstract

This study aimed to elucidate the effects of maternal undernutrition (MUN) on epigenetic modification of hepatic genes in Japanese Black fetal calves during gestation. Using a previously established experimental design feeding the dams with 60% (LN) or 120% (HN) of their global nutritional requirements during the 8.5-month gestational period, DNA methylation in the fetal liver was analyzed with reduced representation bisulfite sequencing (RRBS). The promoters and gene bodies in the LN fetuses were hypomethylated compared to HN fetuses. Pathway analysis showed that the genes with DMR in the exon/intron in the LN group were associated with pathways involved in Cushing syndrome, gastric acid secretion, and aldosterone synthesis and secretion. Promoter hypomethylation in the LN group was frequently observed in genes participating in various signaling pathways (thyroid hormone, Ras/Rap1, PIK3-Akt, cAMP), fatty acid metabolism, and cholesterol metabolism. The promoter hypomethylated genes *ALPL* and *GNAS* were upregulated in the LN group, whereas the promoter hypermethylated genes *GRB10* and *POR* were downregulated. The intron/exon hypomethylated genes *IGF2*, *IGF2R*, *ACAD8*, *TAT*, *RARB*, *PINK1*, and *SOAT2* were downregulated, whereas the hypermethylated genes *IGF2BP2*, *NOS3*, and *NR2F1* were upregulated. Collectively, MUN alters the promoter and gene body methylation of genes associated with hepatic metabolisms (energy, cholesterol, mitochondria) and function, suggesting an impact of altered gene methylation on the dysregulation of gene expression in the fetal liver.

## 1. Introduction

In the early life stages of mammals, the nutrient levels of proteins, amino acids (AAs), carbohydrates, fatty acids (FAs), vitamins, and minerals are crucial for development, growth, and subsequent health maintenance [1]. The deficiency of these nutrients in maternal nutrition during gestation has a severe impact on the fetus, leading to fetal growth restriction (FGR) [1,2]. The malnourished fetal environment does not satisfy the demand for energy and resources to accumulate proteins for skeletal muscle growth in fetal calves [3]. Prolonged nutrition stress due to maternal undernutrition (MUN) impairs not only the growth of a variety of organs, including the skeletal muscles and liver [4,5,6,7,8], but also systemic metabolism by altering the secretion of/sensitivity to glucose, insulin, and insulin-like growth factor-1 (IGF1) [2]. Furthermore, altered glucose homeostasis in MUN-exposed animals often causes long-lasting metabolic diseases in adolescence and adulthood, such as obesity, type II diabetes, and hypertension in humans and animal models [1,9].

The MUN effects are also evident in early epidemiological studies of the Dutch Famine Winter during World War II, which gave rise to the “Developmental Origin of Health and Diseases (DOHaD)” hypothesis, a concept that environmental and nutritional stress experienced by animals in early developmental stages leads to severe chronic diseases in later life [10]. In other words, the systemic metabolism over a life-long period can be programmed by the nutrient levels to which the animals are exposed during early developmental stages via changes in metabolic response to the environmental and nutritional conditions. DNA methylation, the epigenetic mechanism underlying this phenomenon, has been proposed to play a major role in metabolic programming in early life stages [11,12]. However, direct evidence regarding the role of DNA methylation in this programming phenomenon remains limited.

The initial phenotypic effect of MUN on fetuses appears as FGR, which is considered to vary among animal species, gestational stage, restricted nutrients, and period of exposure to low nutrients [13]. The results of FGR differ between animal organs [4,8,14], suggesting that MUN affects fetal DNA methylation differently in different organs. The liver plays an indispensable role in whole-body homeostasis because it plays a role as the center of metabolic regulation [15]; therefore, a functional disturbance of the liver seriously impairs health throughout life. During fetal development, liver growth and function are susceptible to the restriction of global nutrients or calories in pregnant mice [16], rats, guinea pigs, sheep, and cattle [17,18,19,20,21]. In fetal calves, energy metabolism, IGFs, and hormone-mediated gene regulatory networks are disrupted [21]. Moreover, these liver disturbances potentially result in a predisposition of the fetus to systemic homeostatic disorders and subsequent metabolic diseases in later life [11], such as obesity, type II diabetes, and non-alcoholic fatty liver disease (NAFLD) [22]. These observations suggest that the adverse phenotypic effects of MUN in later life are mediated by DNA methylation.

In the offspring of pregnant protein-restricted rat dams, hepatic DNA methylation of peroxisomal proliferator-activated receptor α (*PPARA*) and glucocorticoid receptor (*GR*) is lowered, which is accompanied by transcriptional upregulation [23,24,25]. A low-protein diet fed to dams during gestation alters the gene methylation of insulin-like growth factor 2 (*IGF2*), nuclear receptor 3C1 (*NR3C1*), cytochrome P450-2C34 (*CYP2C34*), and liver X-receptor α (*LXRA*) in mouse, rat, and pig offspring [26,27,28]. Thus, the methylation of these key metabolic genes in the fetal liver is likely altered by aberrant dietary nutrient levels in pregnant dams. Recently, we observed a notable disturbance in gene expression specific to liver functions, such as gluconeogenesis, steroid biosynthesis, glucuronidation, and the urea cycle in bovine MUN fetuses [21]. This suggests that DNA methylation of genes associated with liver-specific functions could be altered by MUN.

The effect of MUN on fetal growth and metabolism is of primal importance in farm animals, leading to the alteration of productive traits, including meat yield and quality. The adverse effects of MUN on animals lead to significant economic losses in the animal industry. Meanwhile, intramuscular triglyceride content is upregulated in the nutrition-restricted fetal skeletal muscles [29]. This indicated that the skeletal muscles of nutrition-restricted fetuses are predisposed to insulin resistance by fetal programming, which can lead to intramuscular fat accumulation in beef, an important attribute for high marbled beef, especially for Wagyu (Japanese Black; JB) cattle. Epigenetic modifications, such as DNA methylation, potentially underlie the MUN effect; therefore, methylated CpG (CG) loci could be potential biomarkers for predicting livestock productivity to select advantageously programmed animals. In addition, farm animals, including sheep, are good models for investigating the impact of MUN on human health and disease because of their similarity to human placental and fetal development compared to rodents [9].

With this background, the present study addressed the effect of global nutrient restriction in pregnant cows on the fetal calf liver epigenetics, focusing on the CG methylation of hepatic genes. Dams of JB cattle were fed 60% (LN) and 120% (HN) of nutritional requirement levels based on protein, fat, and energy contents during the gestation until month 8.5 post-conception, with 120% nutritional level for non-pregnant cows being considered as a control level. This nutritional level meets approximately 100% of the requirements for the maintenance of pregnant JB cows, based on the Japanese Feeding Standard for Beef Cattle (JFSBC) (2008 ed.) [30]. Using this experimental design, we previously observed significant phenotypic differences in various tissues between LN and HN fetuses [3,8,21]. Here, CG methylation of the fetal liver was analyzed by reduced representation bisulfite sequencing (RRBS), the data of which were further subjected to bioinformatics analyses to understand the impact of MUN on CG methylation of genes in the fetal liver.

## 2. Results

### 2.1. Phenotypic Effect of Maternal Nutrient Restriction on Fetuses

The growth of the entire body and liver of fetal calves was affected by MUN. The ratios of body weight (BW) to liver weight in the LN group to those in the HN group were 0.71 and 0.74, respectively, indicating significantly lower BW and liver weight in the LN fetuses compared to the HN fetuses (*p* ≤ 0.05) (Table 1). No significant difference in the percentage of liver weight was observed between groups. MUN markedly decreased liver mass in the LN group.

### 2.2. Distribution of DNA Methylation in LN and HN Fetal Liver Genome

To investigate the effect of MUN on genomic DNA (gDNA) methylation in the fetal liver, we conducted an RRBS analysis of differentially methylated DNA. For downstream analyses of the sequencing, we calculated single-base read coverage and genome coverage distribution, which confirmed that the average base coverage of the genome was more than 10× across the samples. The mapping result of the obtained sequences to the reference genome showed that the total cytosine coverage of the genome was 62.2–81.1 Mb across the samples, whereas the average methylation level of all the cytosines was 6.56–6.61% (Table S1). These results indicated that despite the lower site number, CG was much more methylated than CHG and CHH. In addition, variations in the methylation levels in each genomic region were much higher in the CHG and CHH contexts than in the CG context. Therefore, we focused on methylation levels in the CG for subsequent analyses.

**Figure 1:**
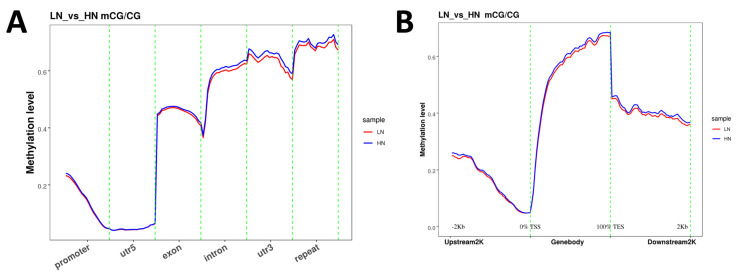
Genomic locus-dependent distribution of CG methylation level in the context of functional genetic elements (**A**) and upstream/downstream 2kb-regions of gene body (**B**). After combining the samples with biological repeats, each region is divided into 20 bins (**A**), or 50 bins (**B**), and the methylation level is calculated in each bin. X-axis: functional genetic elements or regions, y-axis: methylation level. LN: red, HN: blue.

The analysis of methylation levels in each functional genomic region revealed that the methylation in the 5′-untranslated region (UTR5) was at a minimal level (Figure 1A). In contrast, the levels of methylation in the 3′-untranslated region (UTR3) and repeated region were notably higher compared to other regions in both the LN and HN groups. In the exon, intron, UTR3, and repeat regions, the methylation levels were slightly lower in the LN group than in the HN group. Within the gene structure, methylation was at the lowest level at the translation start site (TSS). The methylation level was increased with the distance from the TSS towards upstream or downstream in the coding regions (Figure 1B). The downstream region at a distance of 2 kb from the translation end site (TES) had a lower methylation level compared to TES.

### 2.3. Differential DNA Methylation between LN and HN Fetal Liver

**Figure 2:**
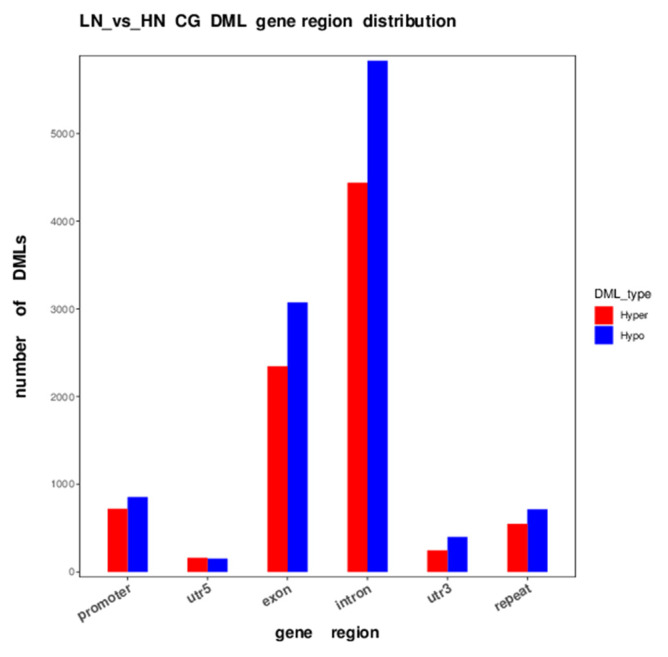
DML distribution in CG context in different functional regions. The x-axis is functional regions; the y-axis is the number of hyper/hypo DMR in each region. Hyper and hypo DMLs in LN fetuses are indicated as red and blue, respectively.

The distribution of the differentially methylated (DM) region (DMR) and CG locus (DML) in each functional genomic region revealed a remarkable trend, where hypomethylation was more predominant than hypermethylation in the promoter, exon, and intron regions of genes in LN fetal liver (Table 2, Figure 2). In particular, the number of hypomethylated CG loci was approximately 1.3-fold higher than that of hypermethylated loci in the entire genome, especially the exon and intron regions (Table 2).

**Figure 3:**
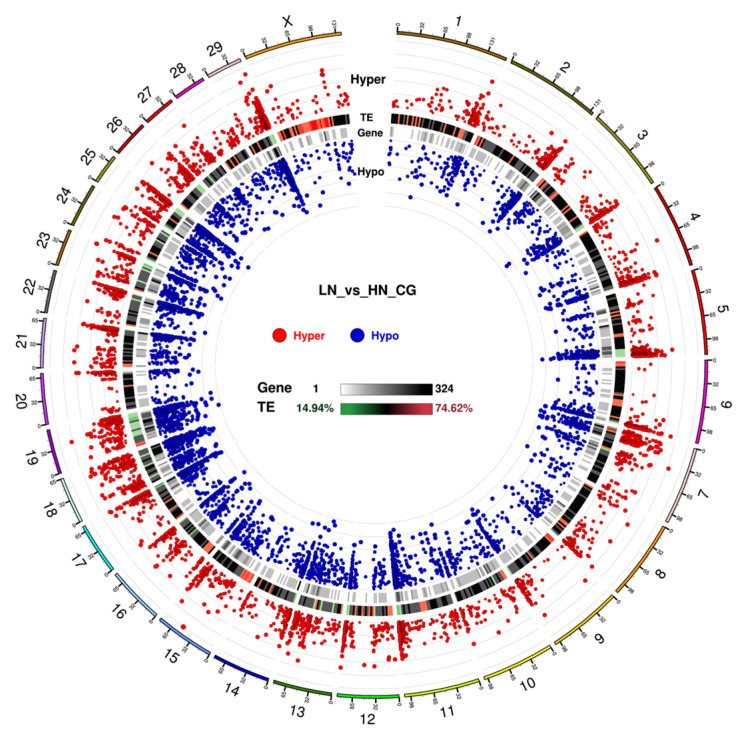
Circos plot for DMR condition in CG context. The circos plot represents (from outside to inside): (1) Hyper DMR statistical value: log5 (|areaStat|). The higher and bigger the point, the larger differences between the two groups. (2) TE, the heatmap of the percentage of repeat elements (if repeats are provided). (3) Heatmap of gene density. (4) Hypo DMR statistical value: log5 (|areaStat|). The higher and bigger the point, the larger differences between the two groups. The number shown in the outside indicates the chromosome number.

In the promoter region, the number of hypomethylated loci was 1.19-fold higher than that of the hypermethylated loci in the LN fetuses. Notably, in the gene body and repeat regions, the ratio of the hypermethylated loci/total hypermethylated loci was similar to the ratio of the hypomethylated loci/total hypomethylated loci, whereas the ratio of hypermethylated loci was slightly different from that of hypomethylated loci in promoter and UTR. The significance and distribution of DMR on specific chromosomes are illustrated in a circus plot (Figure 3). The telomeric region in most of the chromosomes showed an abundance trend in both hyper- and hypo DMR. Meanwhile, chromosomes 18, 19, 23, and 25 were dense with uniformly distributed DMR. Notably, the upstream region, excluding the telomeric region, of chromosome 7 was abundantly hypermethylated and hypomethylated, which was not observed in the other chromosomes.

### 2.4. Kyoto Encyclopedia of Genes and Genomes (KEGG) Pathways Associated with Differentially Methylated Genes

**Figure 4:**
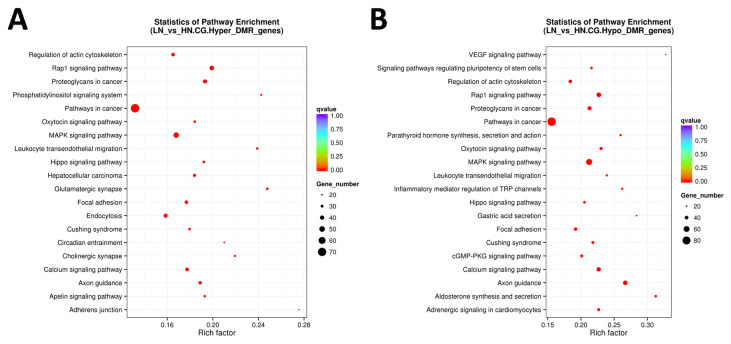
The scattered plot of DMR-related pathways analyzed by KEGG enrichment. (**A**) Genes of all hyper DMR regions, (**B**) genes of all hypo DMR regions. The x-axis represents the Rich factor, and the y-axis represents the pathway name. The size of points stands for DMR-related gene counts, and the colors stand for different q-values ranges.

**Figure 5:**
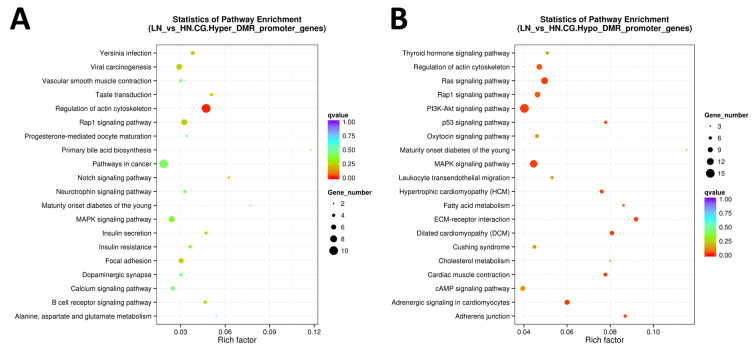
The scattered plot of DMR-related pathways analyzed by KEGG enrichment. (**A**) Genes of hyper DMR in promotor, (**B**) genes of hypo DMR in promotor. The x-axis represents the Rich factor, and the y-axis represents the pathway name. The size of points stands for DMR-related gene counts, and the colors stand for different q-values ranges.

KEGG pathway analysis was performed using all DMR to understand the molecular and cellular events associated with the DM genes (Figure 4 and Figure 5). Regulation of the actin cytoskeleton, Rap1 signaling pathway, oxytocin signaling pathway, MAPK signaling pathway, leukocyte transendothelial migration, Hippo signaling pathway, axon guidance, focal adhesion, calcium signaling pathway, and Cushing syndrome, as well as pathways related to cancer (FDR < 0.0005), were extracted as the top 20 molecular pathways using all DMR-including genes (Figure 4A,B).

When limited to the promoter region, hypermethylated genes were enriched only in the regulation of the actin cytoskeleton (FDR = 0.0083). In contrast, hypomethylated genes were enriched in cellular regulatory pathways, such as Ras signaling pathway, adrenergic signaling in cardiomyocytes, hypertrophic cardiomyopathy (HCM), MAPK signaling pathway, PI3K-Akt signaling pathway, adherens junction, p53 signaling pathway, regulation of actin cytoskeleton, Rap1 signaling pathway, and FA metabolism (FDR < 0.0500; Figure 5A,B). As most of the top 20 pathways were commonly observed in both hyper- and hypomethylated genes when extracted from DM genes in promotor, gene body, and/or UTR, we disregarded the resultant pathways with FDR ≥ 0.001. Of the remaining pathways, after excluding the commonly extracted pathways, six pathways were found to be specific to hypermethylated genes, including mTOR signaling, apelin signaling, and inositol phosphate metabolism (Table 3).

Meanwhile, 26 were identified as pathways specific to hypomethylated genes, including the metabolism of gastric acid secretion and cortisol synthesis/secretion, as well as signaling pathways involving insulin, Ras, PI3K-Akt, neurotrophin, and thyroid hormones.

### 2.5. Genes with Differentially Methylated Loci in Each Genomic Feature

As shown in Figure 2, MUN-altered methylated loci were enriched in the exon, intron, and promoter regions, which raised the possibility of a regional genomic preference for the MUN effect on methylation. Therefore, we focused on the genes with distinct loci in each genomic region. Based on the statistical analysis, significant DML between LN and HN fetuses was extracted. Subsequently, utilizing the extent of the level of CG methylation difference, we identified the top 15 genes that were significantly hypermethylated (Table S2) and hypomethylated (Table S3) in the promoter, exon, and intron regions between the two fetus groups.

Of the promotor hypermethylated genes, lymphocyte-specific protein 1 (*LSP1*), cytochrome p450 oxidoreductase (*POR*), and ankyrin repeat and BTB domain-containing 1 (*ABTB1*) were downregulated in mRNA expression in LN fetal liver at LN/HN ratios of 0.661, 0.873, and 0.796, respectively (*p* < 0.092), in microarray analysis (Table 4). However, no exon/intron-hypermethylated genes showed significant transcriptional differences between LN and HN fetal livers.

G-protein α subunit (*GNAS*) and ribosomal protein S29 (*RPS29*) mRNA expression were upregulated at LN/HN ratios of 1.410 and 1.312, respectively (*p* < 0.060), whereas insulin-like growth factor 2 (*IGF2*) was downregulated (Table 4). The exon/intron-hypomethylated genes unc-13 homolog D (*UNC13D*), sterol *O*-acyltransferase 2 (*SOAT2*), solute carrier family 39 member 11 (*SLC39A11*), *IGF2*, and nth-like DNA glycosylase 1 (*NTHL1*) were downregulated at LN/HN ratios of 0.699, 0.692, 0.798, 0.851, and 0.888, respectively (*p* < 0.090). These results indicated that altered CG methylation in the promoter, exon, and intron regions potentially impact gene expression, with MUN-induced gene-body methylation being particularly associated with the downregulation of hepatic genes.

### 2.6. Alteration in CG Methylation and Gene Expression Is Associated with Energy Metabolism, Steroid Synthesis, and IGF2 Signaling Pathway

During late gestation in the fetal liver, metabolism related to glucose, lipids, tyrosine, steroid hormones, cholesterol synthesis, and reduction–oxidation (redox) processes are affected by the LN status [21]. Moreover, LN conditions altered CG methylation and expression of genes associated with metabolism related to energy homeostasis (*GNAS*), cholesterol (*SOAT2*), insulin/IGF signaling (*IGF2*), oxidative stress (*POR*), and the PI3K/AKT/FOXO1 pathway (*ABTB1*) (Figure 4, Table 4). The expression of these genes can be up- or downregulated by CG methylation in their promoter and/or gene body regions [31]. Accordingly, among significant DM genes participating in IGF signaling, energy homeostasis, redox, and cholesterol metabolisms, we further investigated whether the expression of the relevant genes is linked with the DNA methylation changes or not.

In the quantitative PCR (qPCR) analyses targeting a total of 58 candidate genes, downregulated expression was observed in genes for the regulator downstream of IGF2 signaling, growth factor receptor-binding protein 10 (*GRB10*; promoter hypermethylated), a urea cycle enzyme, tyrosine aminotransferase (*TAT*; intron-hypomethylated), IGF2 receptor (*IGF2R*; intron-hypomethylated), a pivotal signal transducer in lipid metabolism, adenylate kinase 3 (*ADCY3*; promotor hypomethylated), a multifunctional metabolic enzyme, pyruvate carboxylase (*PC*; intron-hypermethylated), and the key mitophagic factor, PTEN-induced kinase 1 (*PINK1*; exon/UTR3-hypomethylated) (Table 5). In addition, the genes associated with lipid homeostasis, retinoic acid receptor β (*RARB*), and acyl-CoA dehydrogenase family member 8 (*ACAD8*) were intron-hypomethylated and downregulated.

Meanwhile, upregulated expression was observed in the genes encoding a NO-controlling enzyme, cystathionine γ-lyase (*CTH*; intron-hypomethylated), a repressor of gluconeogenesis and lipogenesis, C-terminal binding protein 2 (*CTBP2*; intron-hypomethylated), an energy homeostasis regulator, phosphatidylinositol 3-kinase regulatory subunit 1 (*PIK3R1*; exon/UTR3-hypomethylated), and tissue-nonspecific alkaline phosphatase (*ALPL/TNAP*; promoter hypomethylated). The intron-hypermethylated genes for angiogenesis/blood pressure regulator, nitric oxide synthase 3 (*NOS3*), a repressor of hormone-induced receptor responses, nuclear receptor subfamily 2 group F member 1 (*NR2F1*/*COUP-TF1*), and IGF2 mRNA-binding protein 2 (*IGF2BP2/IMP2*) were also upregulated. Thus, the alterations in methylation patterns in these genes indicated that MUN impacts CG methylation of genes associated with IGF2 signaling, glucose and lipid homeostasis, oxidative stress, and haptic key metabolisms.

## 3. Discussion

### 3.1. Impact of MUN on the Hepatic Genome and Molecular Networks in Fetal Calves

In the present study, global maternal undernutrition from the early to late gestation period altered DNA methylation in fetal calf liver. The LN fetal liver had a lower average weight than the HN fetal liver, indicating the severity of the impact of low maternal dietary nutrition on the fetal calf liver. The impact on liver growth might be attributed to the altered methylation of *IGF2* and downstream signaling genes. The RRBS results revealed that the number of hypomethylated CG sites in the genomic DNA of the LN fetal liver was higher than that in the hypermethylated CG sites across the promoter, UTR5, exon, intron, UTR3, and repeat regions, indicating that hypomethylated loci in these gene features were more abundant in LN fetuses than in HN fetuses. Additionally, the DMLs were abundant prominently in the exon and intron regions and were frequently located in proximity to the telomeric end of each chromosome, with some exceptions of the broadly distributed abundance on chromosomes 13, 18, 19, and 25 and a localization near the centromeric region of chromosome 7, as shown in Figure 3. In human chromosomes, DNA methylation is dense in the proximity of telomeres [32]; however, the reason why DMLs are dense in bovines 13, 18, 19, and 25 remains unclear. This trend also suggests that these genomic regions are abundant with genes that are sensitive to MUN impact, although only a few genes are known to be associated with these regions to date.

RRBS, followed by DML analyses, revealed that the number of hypomethylated CG loci in the promoters, exons, introns, and UTR3 regions was higher than that of hypermethylated loci. It is well established that hypermethylation of promoter CpG islands exerts a suppressive effect on gene expression [31]. In addition, in most cases, the exon and intron regions positively affect gene expression, except for the first exon and intron in some cases [33,34]. Hypomethylation is also predominant in the UTR3 region of genes that are expressed [33]. In this study, we identified essential metabolic genes such as *GNAS*, *SOAT2*, *ADCY3*, *PIK3R1*, *TAT*, *ACAD8*, and *PINK1*, as the genes hypomethylated in promotor and gene body in LN fetal liver. Based on the KEGG pathway analysis using the hypomethylated genes, we extracted the relevant pathways regarding vascular endothelial growth factor (VEGF), cGMP, gastric acid, phospholipase D, cortisol, insulin, PI3K-Akt, and thyroid hormone (Table 3). These results suggest the crucial impact of MUN on the fetal liver via DNA hypomethylation.

Taking a closer look at hyper/hypomethylation in the promoter, gene body, and UTRs, 11 pathways/metabolisms were listed as common to both hyper- and hypomethylated genes, including signaling pathways of phosphatidylinositol, Rap1, oxytocin, and calcium. These signaling pathways play a key role in the regulation of various fundamental metabolic pathways in the liver, as evidenced by their influence on the regulation of insulin and glucagon [35,36,37]. One of the representative terms in the pathway analysis, Cushing’s syndrome, is a common metabolic syndrome caused by prolonged exposure to excess cortisol. Chronically elevated glucocorticoid (GC) levels result in hepatic steatosis, insulin resistance, visceral obesity, muscle myopathy, hypertension, and symptoms similar to those of metabolic syndrome [38]. Maintenance of the relevant metabolism is mediated by cAMP signaling [39], which plays multiple roles in hepatic autophagy and glucose, lipid, and cholesterol metabolism [40], cooperatively with other signaling pathways. Accordingly, the molecular network underlying Cushing’s syndrome is likely common to the mechanism where CG methylation changes in the LN fetal liver affect hepatic metabolisms.

The KEGG pathways specific to hypermethylation or hypomethylation were extracted, resulting in a 4.3-fold greater number of pathways from hypomethylated genes compared to hypermethylated genes in LN fetuses. This indicates that metabolisms in the fetal calf liver may be modified by hypomethylation rather than hypermethylation under MUN impact. Hypermethylation-associated pathways in LN fetuses included apelin signaling, mTOR signaling, and inositol phosphate metabolism, which are responsible for lipid metabolism and autophagy in the liver [35,36,41]. The hypomethylation-associated pathways included signaling pathways and metabolisms that are essential for hepatic angiogenesis, gastric acid secretion, adaptive nutrient homeostasis, and hormonal growth regulation [40,42,43], such as insulin/IGF signaling and PI3K-Akt signaling (Table 3). Disruption of any of the above-mentioned pathways/metabolisms results in hepatic steatosis, insulin resistance, fibrosis, and/or cirrhosis [35,36,41].

Intriguingly, promoter hypomethylated genes in the LN fetal liver were found to be significantly associated with essential hepatic metabolisms, such as cholesterol metabolism and FA metabolism. The number of significant molecular events associated with promoter hypomethylated genes was significantly higher compared to hypermethylated genes, which was similar to the differences between hypermethylation and hypomethylation in the gene body. Thus, MUN during gestation in cattle has an epigenetic impact on fetal liver growth and function via hypomethylation of the hepatic essential genes in both promotor and gene body, rather than by hypermethylation.

The present bioinformatics results suggest that the MUN of pregnant dams predisposes the fetal calf liver to metabolic disorders, which can lead to prolonged disruption in energy and cholesterol metabolism in the offspring. Notably, the animals used in this study showed that MUN also altered the metabolite content and gene expression associated with the urea cycle, glucose and lipid metabolism, FA oxidation, cholesterol, and bile acid metabolism, steroid hormone synthesis, and sphingolipid metabolism in our previous study using metabolomic and transcriptomic approaches [21]. Gene expression analyses revealed that genes associated with ketogenesis (*HMGCS2*), gluconeogenesis (*G6PC*, *PCK1*), steroid hormone synthesis (*FDPS*, *HSD11B1*), urea cycle (*ASS1*, *CPS1*), redox processes (*SAO*, *PIPOX*), and FA metabolism (*ADH4*, *EHHADH*) were downregulated, indicating that these metabolic pathways were suppressed in the fetal liver. Collectively, the suppressed metabolic activities may be the result of alterations in DNA methylation of the relevant genes identified in this study.

### 3.2. Hyper/Hypomethylated Genes Essential for Hepatic Metabolisms and Function

Functions of the top DML genes and KEGG pathways indicated that genes with altered methylation were associated with IGF signaling, glucose metabolism, FA oxidation, cholesterol metabolism, steroid hormone synthesis, autophagy, and angiogenesis. Among these, several DM genes showed a trend of changes in transcription, as well as CG methylation (Table 4 and Table 5).

**Figure 6:**
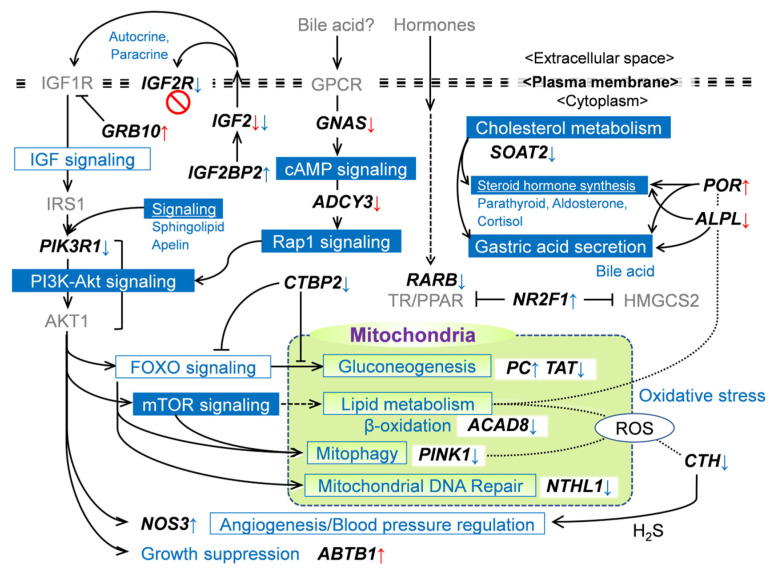
Hypothetic scheme of DM gene-associated molecular regulatory networks and metabolisms in MUN fetal liver. Up- and downward arrows indicate hyper- and hypomethylation in promotor (red) or gene-body (blue), respectively. The pathway/metabolism boxes filled in blue are significantly extracted as relevant to DM genes. Grayed genes are not determined in this study. Solid and chain lines indicate direct and indirect regulations and indirect relationships, respectively.

The insulin/IGF growth factor axis is a key regulatory endocrine factor in pre-and postnatal growth [44]. Among the DM genes associated with IGF signaling, IGF2BP2 and IGF2R play contrasting roles, with IGF2BP2 exerting a positive influence and IGF2R exerting a negative influence on IGF2 signaling [45,46,47] (Figure 6). GRB10 is a negative regulator of receptor-type tyrosine kinases, including IGF1R, a major receptor for IGF2, although the effect of GRB10 on IGF signaling is controversial [48]. Intriguingly, the expression of these IGF signaling molecules was modulated in a direction to amplify the IGF2 signal in the LN fetal liver. *GRB10* was downregulated, which could be due to the hypermethylation of its promoter. In contrast, *IGF2* expression was negatively modulated in LN fetuses, although its promoter was hypomethylated. One possible explanation for the inconsistency in *IGF2* expression compared to other activated IGF signaling genes is that *IGF2* may be downregulated at the transcriptional level by another repressor.

Our RRBS analyses revealed that the PI3K-Akt signaling, phosphatidylinositol signaling, and inositol phosphate pathways were also different between the LN and HN groups. The PI3K-Akt signaling pathway plays a central role in the physiological adaptation to the cellular environment and nutrition, especially in energy homeostasis mediated by insulin signaling [49] (Figure 6). The *PIK3R1*, the key regulator of the PI3K-Akt signaling pathway, is hypomethylated in exon/UTR3 and upregulated in LN fetuses. Despite the unknown relationship between hypermethylation and upregulated expression, hypermethylation may affect the expression or alternative splicing of this gene. The upregulation of *PIK3R1* could support signals from upstream pathways derived from the IGF1R.

In addition, the PI3K-Akt signaling pathway mediates hormone-induced cAMP signaling, including *ADCY3*, as the central player in energy homeostasis [50,51], and mTORC1/2 stimulation via Rap1 activation [36]. GSα encoded by the *GNAS* gene is the stimulatory subunit that can activate ADCY3 to promote the production of cAMP, disruption of which results in obese and insulin-resistant phenotypes [50,52]. In this context, hypomethylation of *ADCY3* and *GNAS* promoters was reasonable under the MUN environment because promoter hypomethylation would result in the activation of gene expression and subsequent energy metabolic adaptation via downstream signaling. In LN fetuses, however, *ADCY3* expression was downregulated in a similar manner to the *IGF2* expression, which may be attributed to negative regulation exerted by certain repressors.

The PI3K-Akt pathway downstream genes associated with lipid metabolism and gluconeogenesis were hypermethylated or hypomethylated by MUN. Some of these genes were activated by growth factor (GF) and/or hormone signals via downstream pathways, such as FOXO and mTOR signaling (Figure 6). *POR* and *ACAD8* were promoter hypermethylated and intron-hypomethylated, respectively, and both were downregulated in LN fetuses. In hepatic lipid metabolism, *POR* plays a central role in catabolic activity by metabolizing xenobiotics and steroid hormones [53,54]; additionally, it plays a key role in cholesterol [55] and steroid synthesis [56]. *ACAD8* is a gene encoding isobutyryl-CoA dehydrogenase, the dysregulation of which leads to hepatic steatosis via mitochondriopathy [57]. Regulated by acyl-CoA and NADH, CTBP2 reciprocally modulates lipid metabolism and FOXO-mediated gluconeogenesis by acting as a transcriptional repressor [58]. *CTBP2* is hypomethylated in its intron, but its expression is downregulated in LN fetuses, which may be affected by other transcriptional modulators. These findings suggest that MUN modulates hepatic lipid metabolism via altered DNA methylation of *PIK3R1* and mTOR signaling genes, as well as *POR* and *ACAD8* in fetal calves. In addition, *PC* and *TAT*, mitochondrial gluconeogenic genes that respond to insulin, glucocorticoids, and/or glucagon [59,60,61,62], were downregulated with altered DNA methylation in LN fetuses. All the genes involved in lipid metabolism and gluconeogenesis were downregulated. Collectively, these gene expressions may be modulated by DNA methylation changes, thereby restricting glucose production and FA oxidation in LN fetal liver.

Mitochondrial activities and viability may be compromised in LN fetuses, as indicated by the downregulation of *PINK1*, a key player in mitophagy [63] (Figure 6). In the calf liver, low levels of *PINK1* expression are associated with reactive oxygen species (ROS) overproduction in cows with a fatty liver, which causes ROS overproduction and lipid accumulation in hepatocytes [64]. The low level of mitophagy marker indicated impaired mitophagy and its potential impact on the stability of mitochondrial DNA. This was further supported by the downregulation of *NTHL1*, a mitochondrial DNA glycosylase, which was downregulated due to MUN. Low *PINK1* expression may be induced by mTOR signaling activity originating from upstream GF and hormone signals [65]. The notable abundance of DM hepatic genes encoding key mitochondrial metabolic enzymes (*PC*, *TAT*, *ACAD8*, *PINK1*, and *NTHL1*) is a noteworthy finding that highlights the central role of mitochondria in metabolic adaptations in response to nutritional stress in the fetal liver. However, the mechanisms underlying the transduction of nutritional signals into alterations in nuclear DNA methylation remain unclear.

Though the alterations in methylation differed, *CTH* and *NOS3* were upregulated in LN fetuses. *CTH*-deficient mice exhibited elevated oxidative stress and impaired eNOS, the *NOS3* product, resulting in reduced NO levels [66]. CTH generates H_2_S, which, in turn, controls endothelial NO bioavailability and blood pressure [67] and protects against oxidative stress [68]. Thus, *CTH* and *NOS3* cooperatively fine-tune NO levels and regulate blood pressure and/or angiogenesis [69,70,71]. Altered methylation of these genes may contribute to the maintenance of angiogenesis and blood pressure in fetuses with LN.

*POR*, a hypermethylated gene in LN fetuses, plays an essential role in bile production [53,56] and steroid synthesis [56,72]. Despite the functional importance, *POR* expression was suppressed in MUN condition, which might be due to the reduced demand for its activity in steroid metabolism, as indicated by our previous study [21]. In addition, a key gene for the hepatic cholesterol-esterifying enzyme *SOAT2* was downregulated due to MUN and hypomethylated in its exon and intron. A deficiency in its activity results in decreased retention of triglycerides (TG) and cholesteryl esters in the liver [73,74]. Furthermore, *ALPL*, a key player in bile secretion in hepatocyte and cholangiocyte membranes facing bile canaliculi and ducts [75], was hypomethylated and upregulated. The *POR* activity for steroid hormone synthesis was likely restricted in LN fetuses, although *ALPL* expression may promote bile secretion, particularly in LN fetuses. Taken together, altered methylation of genes involved in cholesterol metabolism suggests that less bile secretion and lipid accumulation in the liver are prioritized in the LN fetal liver. This response may be crucial for the physiological adaptation of dams to adverse nutritional stress.

MUN-induced *RARB* downregulation may affect the quiescence of hematopoietic stem cells (HSC) in the fetal liver, where HSC proliferation and development during normal fetal life are maintained [76]. *RARB* was hypomethylated in its intron and downregulated in LN fetuses. The role of *RARB* as a regulator of the protein translation rate and ROS levels in dormant HSC has been recently reported [77]. This is important for maintaining quiescence in HSCs. In addition, the maintenance of HSC was found to be dependent on 4-oxo-retinoic acid (RA), a potent agonist that specifically targets RARβ [78,79], as well as the expression of RARβ itself [80]. Hypomethylation of *RARB* may regulate HSC quiescence in the LN liver and predispose the cells to an activated state in the offspring. The transcriptional repressor COUP-TF1 (*NR2F1* product) regulates the expression of hepatic genes, including mitochondrial HMG-CoA synthase (*HMGCS2*), phosphoenolpyruvate carboxykinase (*PCK1*), and angiotensinogen, cooperatively with hepatocyte nuclear factor 4 (HNF-4) [81,82]. This repressor was hypermethylated with concomitant upregulation in the present study. Coincidently, *HMGCS2* expression is downregulated in the LN fetal liver [21], which could be the result of hypermethylation and upregulation of the repressor *NR2F1*. Further, *NR2F1* is also thought to repress nuclear receptors, including RARs, thyroid hormone receptors (TR), and peroxisome proliferator-activated receptors (PPAR) [83]. Accordingly, the upregulation of *NR2F1* may have a significant impact on the hormonal induction of metabolic disorders in LN fetuses and postnatal life.

## 4. Materials and Methods

### 4.1. Animals and Dietary Treatment

Eleven multiparous JB cows (initial BW 488 ± 9.6 kg) fed at the Iriki farm of Kagoshima University and the farm of the Western Region Agricultural Research Center (NARO) were managed and used, and fetuses were obtained as previously described [21]. Briefly, individual diets were designed for pregnant JB cows to meet 60% or 120% of the energy requirement and other nutrients based on the standard diet calculated for BW before pregnancy according to the Japanese Feeding Standard for Beef Cattle (2008 ed.) (NARO) [30]. The diet composition was designed as previously described [21]. Cows were randomly assigned to LN (*n* = 5) or HN (*n* = 6) diet groups and fed their respective diets during gestation. Fetuses were obtained from cows via cesarean section. The animals were maintained in accordance with the Guide for the Care and Use of Experimental Animals. The experimental design was approved by the Animal Care and Use Committee of Kagoshima University (#A18007).

### 4.2. Sample Collection

The fetuses were injected with lidocaine (AstraZeneca, Osaka, Japan) into the jugular vein and euthanized by exsanguination at day 260 ± 8.3 of gestation. From the dissected fetal carcass, liver samples were obtained, a portion of which were frozen using liquid nitrogen for DNA preparation or soaked in RNA*later*^®^ (Thermo Fisher Scientific, Tokyo, Japan) for gene expression analysis, and stored at −80 °C until used for subsequent analyses. Among the fetuses from the cows, the three with the highest BW and three with the lowest BW were selected as HN and LN fetuses, respectively, for subsequent comparisons, including differential methylation analyses.

### 4.3. Genomic DNA Preparation

The genomic DNA samples were extracted using a QIAamp DNA Mini Kit (Qiagen, Hilden, Germany), according to the manufacturer’s protocol for the preparation of RRBS samples. The prepared gDNAs were quantified using a Quant-iT dsDNA High-Sensitivity Assay Kit (Life Technologies, Tokyo, Japan) and a VersaFluor spectrometer (Bio-Rad, Hercules, CA, USA). The quality of the gDNA samples was confirmed by resolution in 1% agarose gel electrophoresis based on the extent of DNA fragmentation and RNA contamination.

### 4.4. Reduced Representation Bisulfite Sequencing (RRBS)

The qualified samples were digested using the methylation-insensitive restriction enzyme *Msp*I, followed by dA-tailing and ligation to sequencing adaptors. DNA fragments with insertion lengths ranging from 40 bp to 220 bp were selected by isolating specific gel bands. The selected DNA fragments were treated with bisulfite using the EZ DNA Methylation Gold Kit (Zymo Research, Orange, CA, USA), after which the fragments were amplified using PCR. The constructed library was quantified with a Qubit 2.0 Fluorometer (Thermo Fisher Scientific), followed by accurate quantification of the library concentration by Q-PCR (effective library concentration > 2 nM) to ensure library quality. Sequencing was performed for different libraries according to the concentration and data demand on the Illumina HiSeq/NovaSeq platform (Illumina, San Diego, CA, USA). The raw reads obtained in FASTQ format were filtered to remove contaminated adapter sequences, and low-quality reads using Trimmomatic-0.36 (Illumina). Bisulfite-treated reads were aligned to a reference genome (ARS-UCD1.2/bosTau9, April 2018), and methylated CG calling and annotations of the promoter, gene body, and CpG islands were performed by Bismark [84] using the deduplicate_bismark and bismark_genome_preparation commands.

### 4.5. Analyses of DMR and DML

To identify the true methylated sites, methylated and unmethylated counts at each site from the Bismark output were tested using a binomial distribution. The following set of thresholds was set in the analysis process to find accurate methylated sites [85,86]: (1) the sequencing depth was greater than or equal to five, and (2) the q-value (FDR) was less than or equal to 0.01. For the methylated sites, the methylation level was calculated using the following formula: ML = mC/(mC + umC), where ML represents the methylation level and mC and umC represent the number of methylated and unmethylated cytosines, respectively.

DMR and DML were analyzed using the DSS-single (DSS_2.12.0) pipeline [87,88], which provides the average methylation level of the DMR to draw the methylation level distribution as a heatmap of hierarchically clustered regions. Considering the spatial correlation of the DMR, smoothing was applied during the analysis. For DMR distribution in the genomic context, hyper- and hypomethylated regions were statistically analyzed for promoters, exons, introns, repeats, TSS, TES, and other regions. DMR distribution in the genome and significant analysis were also illustrated by a Circos plot using circos-0.62-1 [89]. DMR screening was controlled with cut-off values of minimum length (50 bp), CG content (three sites), and adjusted *P*-value (false discovery rate; FDR) for comparison between the groups (FDR = 0.10). In the DMR and DML analyses, differences were considered significant at FDR ≤ 0.10.

### 4.6. Pathway Enrichment Analyses for Hyper- or Hypo-Methylated Genes

Kyoto Encyclopedia of Genes and Genomes (KEGG) pathway enrichment analysis (http://www.kegg.jp/, accessed on 26 March 2023) [90], KOBAS (2.0) [91] was used. The analysis was performed based on the distribution of DMR in the genome of the promoter, gene body region (from TSS to TES), and overlapping genes with DMR in CG dinucleotide sequences. In this analysis, a hypergeometric test was used to identify the biological metabolism and pathways that were significantly enriched in DMR-related genes compared to the entire genomic background. An adjusted *p*-value < 0.10 (FDR) was regarded as significant.

### 4.7. Microarray Analysis

The fetuses analyzed were those with the lowest BW in the LN group (four animals) and those with the highest BW in the HN group (four animals). Total RNA samples from four fetuses in the HN and LN groups were subjected to Bovine (v2) Gene Expression 4× 44 K Microarray (Agilent Technologies, Waldbronn, Germany). The details are described in a previous study [21]. Signals from the hybridized probes were detected using an Agilent microarray scanner (Agilent). Results were normalized using the quantile method in GeneSpring GX (Agilent).

### 4.8. Gene Expression Analysis in Quantitative PCR

The total RNA extraction from LN and HN fetal livers and the cDNA synthesis were performed as described previously [21]. To test the expressions of the differentially methylated (DM) genes, qPCR was performed using the cDNA templates of liver samples. The sequences of the primers used for qPCR are provided in Table S4. The ribosomal protein lateral stalk subunit P0 (*RPLP0*) was used as an internal control. Melting curve analysis was performed to confirm the specificity of amplification reactions.

### 4.9. Statistical Analyses

To compare the gene expression between LN and HN groups, the BW and liver weight were analyzed by the two-sided Student’s *t*-test. The PCR data were analyzed by the one-sided Student’s *t*-test, based on the trend of gene expression in previous microarray analysis [21]. Differences were considered significant at *p* ≤ 0.05 or a trend at *p* ≤ 0.10 for the microarray and PCR results.

## 5. Conclusions

DNA methylation status was altered in the genes associated with fundamental hepatic metabolism in the fetal calf liver at the late gestational stage when maternal nutrient levels were globally restricted. DNA methylation of *IGF2* and critical metabolic genes changed concomitantly with liver growth retardation, resulting in a reduced fetal liver mass. The modification in gene expression, regulatory pathways, and metabolism in the fetal calf liver was predominantly regulated by hypomethylation rather than hypermethylation under MUN conditions. DMLs for both hyper- and hypomethylation were involved in essential signaling pathways for growth and liver metabolism, such as IGF2, mTOR, PI3K-Akt, cAMP, and FOXO signaling. Methylation-altered *ADCY3*, *GNAS*, and *PIK3R1* may have critical roles in gene activation and subsequent cellular metabolic adaptation via downstream signaling. It is most likely that DNA methylation in the LN fetal liver downregulated the expression of genes involved in lipid metabolism, gluconeogenesis, cholesterol metabolism, and mitochondrial viability. Thus, MUN treatment had a significant impact on the DNA methylation of hepatic genes, hepatic metabolism, and functions of the fetal calf liver. These alterations may affect the development of metabolic disorders during the later life of the offspring.

## Figures and Tables

**Table 1:** Effect of maternal nutritional restriction on fetal phenotypes.

Trait	HN (*n* = 3)		LN (*n* = 3)		*p*-Value
	Mean	SEM ^1^	Mean	SEM ^1^	
Age (d)	261.33	2.03	257.67	0.88	0.173
BW (kg)	33.31	0.42	23.5	1.72	0.005
Liver (g)	659.37	4.49	485.15	20.98	0.001
%BW	1.98	0.01	2.08	0.15	0.529

^1^ SEM: standard error of the mean.

**Table 2:** The number of differentially methylated CG loci in LN fetal liver for each genomic feature ^1^.

Genomic Region	Hyper		Hypo	
	Loci	%	Loci	%
Promoter	718	8.50	854	7.75
UTR5	159	1.88	150	1.36
Exon	2344	27.73	3075	27.90
Intron	4440	52.53	5832	52.92
UTR3	244	2.89	397	3.60
Repeat	547	6.47	712	6.46
Total	8452	100.00	11,020	100.00

^1^ The number of differentially methylated loci in the LN fetuses compared to HN fetuses was separately indicated as hypermethylation or hypomethylation type.

**Table 3:** Significant KEGG pathways specific to hyper- and hypomethylated genes in promotor, gene body, and/or UTRs ^1^.

Type of Difference	KEGG Pathway	FDR
Hypermethylation		
	Apelin signaling pathway	0.000156
	mTOR signaling pathway	0.000525
	AGE-RAGE signaling pathway in diabetic complications	0.000548
	Inositol phosphate metabolism	0.000651
	Wnt signaling pathway	0.000862
	Amoebiasis	0.000884
Hypomethylation		
	Adrenergic signaling in cardiomyocytes	0.000008
	Inflammatory mediator regulation of TRP channels	0.000011
	Parathyroid hormone synthesis, secretion, and action	0.000012
	VEGF signaling pathway	0.000029
	cGMP-PKG signaling pathway	0.000043
	Gastric acid secretion	0.000043
	Signaling pathways regulating pluripotency of stem cells	0.000046
	Platelet activation	0.000066
	Phospholipase D signaling pathway	0.000142
	Cortisol synthesis and secretion	0.000167
	GnRH signaling pathway	0.000167
	Type II diabetes mellitus	0.000167
	Relaxin signaling pathway	0.000184
	Sphingolipid signaling pathway	0.000275
	Insulin signaling pathway	0.000306
	Yersinia infection	0.000370
	Ras signaling pathway	0.000370
	Dopaminergic synapse	0.000390
	Human papillomavirus infection	0.000390
	Vascular smooth muscle contraction	0.000417
	Insulin resistance	0.000444
	Tight junction	0.000552
	PI3K-Akt signaling pathway	0.000615
	Neurotrophin signaling pathway	0.000662
	Human cytomegalovirus infection	0.000767
	Thyroid hormone signaling pathway	0.000988

^1^ KEGG pathways extracted from DM genes in the LN fetuses were separately indicated as hypermethylation or hypomethylation type.

**Table 4:** Hyper- and hypomethylated genes accompanied by transcriptional changes.

Chr. No. ^1^	Position	Methylation Difference	Gene Name	Genomic Region	FDR	Microarray	
						LN/HN ^2^	*p*-Value
29	49,625,542	0.425	LSP1	promoter	0.017	0.661	0.010
25	34,197,023	0.342	POR	promoter	0.059	0.873	0.056
22	59,839,258	0.335	ABTB1	promoter	0.003	0.796	0.092
13	57,484,897	−0.147	GNAS	promoter	0.002	1.410	0.003
10	42,629,559	−0.366	RPS29	promoter	0.001	1.312	0.060
19	55,784,207	−0.394	UNC13D	exon	0.000	0.699	0.003
5	26,877,080	−0.456	SOAT2	intron	0.003	0.692	0.031
5	26,877,156	−0.470	SOAT2	exon	0.001	0.692	0.031
19	58,319,907	−0.566	SLC39A11	intron	0.001	0.798	0.090
29	49,403,320	−0.571	IGF2	intron	0.000	0.851	0.056
29	49,403,299	−0.571	IGF2	promoter	0.001	0.851	0.056
25	1,592,534	−0.594	NTHL1	intron	0.000	0.888	0.040

^1^ Chr. no.: chromosome number. ^2^ LN/HN: ratio of gene expression of LN to HN.

**Table 5:** Hyper- and hypomethylated genes associated with hepatic growth, energy, cholesterol, and mitochondrial metabolisms.

Chr. No. ^1^	Position	Methylation Difference	Gene Name	Genomic Region	FDR	PCR	
						LN/HN ^2^	*p*-Value
4	5,187,337	0.358	GRB10	promoter	0.025	0.341	0.074
18	39,490,208	−0.197	TAT	intron	0.023	0.554	0.043
9	96,221,893	−0.436	IGF2R	intron	0.000	0.555	0.058
11	74,335,256	−0.205	ADCY3	promoter	0.050	0.581	0.086
27	40,235,976	−0.226	RARB	intron	0.065	0.592	0.076
29	44,889,960	0.191	PC	intron	0.078	0.623	0.070
2	131,986,576	−0.382	PINK1	exon/utr3	0.000	0.717	0.049
15	83,530,698	−0.588	ACAD8	intron	0.000	0.797	0.100
3	74,775,896	−0.423	CTH	intron	0.067	1.488	0.059
4	113,578,206	0.286	NOS3	intron	0.006	1.751	0.079
7	93,056,076	0.205	NR2F1	intron	0.000	2.035	0.097
20	11,399,276	−0.489	PIK3R1	exon/utr3	0.020	2.043	0.027

^1^ Chr. no.: chromosome number. ^2^ LN/HN: ratio of gene expression of LN to HN.

## Data Availability

Array data were deposited in the National Center for Biotechnology Information (NCBI) Gene Expression Omnibus (GEO) database and are accessible through GEO Series accession number GSE191179 (http://www.ncbi.nlm.nih.gov/geo).

## Supplementary Materials

The following supporting information can be downloaded at: https://www.mdpi.com/article/10.3390/ijms241310682/s1.
